# Effect of SARS-CoV-2 Infection in Pregnancy on Maternal and Neonatal Outcomes in Africa: An AFREhealth Call for Evidence through Multicountry Research Collaboration

**DOI:** 10.4269/ajtmh.20-1553

**Published:** 2020-12-28

**Authors:** Jean B. Nachega, Nadia A. Sam-Agudu, Samantha Budhram, Taha E. Taha, Valerie Vannevel, Priya Somapillay, Daniel Katuashi Ishoso, Michel Tshiasuma Pipo, Christian Bongo-Pasi Nswe, John Ditekemena, Birhanu T. Ayele, Rhoderick N. Machekano, Onesmus W. Gachuno, John Kinuthia, Nancy Mwongeli, Musa Sekikubo, Philippa Musoke, Evans Kofi Agbeno, Lawal W. Umar, Mukanire Ntakwinja, Denis M. Mukwege, Emily R. Smith, Eduard J. Mills, John Otokoye Otshudiema, Placide Mbala-Kingebeni, Jean-Marie N. Kayembe, Don Jethro Mavungu Landu, Jean-Jacques Muyembe Tamfum, Alimuddin Zumla, Eduard J. Langenegger, Lynne M. Mofenson, for the AFREhealth COVID-19 Research Collaboration Working Group

**Affiliations:** 1 Department of Medicine, Stellenbosch University, Cape Town, South Africa;; 2 Department of Epidemiology, Infectious Diseases and Microbiology, Center for Global Health, University of Pittsburgh, Pittsburgh, Pennsylvania;; 3 Department of Epidemiology, Johns Hopkins Bloomberg School of Public Health, Baltimore, Maryland;; 4 Department of International Health, Bloomberg School of Public Health, Johns Hopkins University, Baltimore, Maryland;; 5 International Research Center of Excellence, Department of Pediatrics and Institute of Human Virology Nigeria, Abuja, Nigeria;; 6 Department of Pediatrics, Institute of Human Virology, University of Maryland School of Medicine, Baltimore, Maryland;; 7 Department of Paediatrics and Child Health, University of Cape Coast School of Medical Sciences, Cape Coast, Ghana;; 8 Department of Obstetrics and Gynecology, University of KwaZulu Natal, Durban, South Africa;; 9 UP/SAMRC Maternal and Infant Health Care Strategies Unit, Department of Obstetrics and Gynecology, Kalafong Hospital, University of Pretoria, Pretoria, South Africa;; 10 Maternal Foetal Medicine, Steve Biko Hospital, University of Pretoria, Pretoria, South Africa;; 11 Department of Community Health, School of Public Health, University of Kinshasa, Kinshasa, Democratic Republic of the Congo;; 12 Faculty of Public Health, Université Moderne de Kinkole, Kinshasa, Democratic Republic of Congo;; 13 Department of Public Health, Faculty of Medicine, Centre Interdisciplinaire de Recherche en Ethnopharmacologie, Université Notre-Dame du Kasayi, Kananga, Democratic Republic of Congo;; 14 Division of Epidemiology and Biostatistics, Department of Global Health, Faculty of Medicine and Health Sciences, Stellenbosch University, Cape Town, South Africa;; 15 Department of Obstetrics and Gynaecology, University of Nairobi, Nairobi, Kenya;; 16 Department of Research, Department of Reproductive Health, Kenyatta National Hospital, Nairobi, Kenya;; 17 Department of Obstetrics and Gynaecology, School of Medicine, College of Health Sciences, Makerere University, Kampala, Uganda;; 18 Department of Paediatrics and Child Health, School of Medicine, College of Health Sciences, Makerere University, Kampala, Uganda;; 19 Department of Obstetrics and Gynecology, Cape Coast Teaching Hospital, University of Cape Coast, Cape Coast, Ghana;; 20 Department of Pediatrics, College of Health Sciences, Ahmadu Bello Teaching Hospital, Ahmadu Bello University, Zaria, Nigeria;; 21 Gynaecology and General Surgery, Panzi General Referral Hospital, Bukavu, Democratic Republic of the Congo;; 22 Department of Global Health, Milken Institute School of Public Health, George Washington University, Washington, District of Columbia;; 23 Department of Health Research Evidence and Impact, Faculty of Health Sciences, McMaster University, Hamilton, Canada;; 24 Epidemiological Surveillance Team, COVID-19 Response, Health Emergencies Program, World Health Organization, Kinshasa, Democratic Republic of the Congo;; 25 Department of Medicine, Faculty of Medicine, University of Kinshasa, Kinshasa, Democratic Republic of the Congo;; 26 Department of Medical Microbiology and Virology, Faculty of Medicine, National Institute of Biomedical Research (INRB), University of Kinshasa, Kinshasa, Democratic Republic of the Congo;; 27 Division of Infection and Immunity, Department of Infection, Centre for Clinical Microbiology, University College London, London, United Kingdom;; 28 National Institute for Health Research Biomedical Research Centre, University College London Hospitals, London, United Kingdom;; 29 Department of Obstetrics and Gynecology, Tyberberg Teaching Hospital, Stellenbosch University Faculty of Medicine and Health Sciences, Cape Town, South Africa;; 30 Elizabeth Glaser Pediatric AIDS Foundation, Washington, District of Columbia

## Abstract

In the African context, there is a paucity of data on SARS-CoV-2 infection and associated COVID-19 in pregnancy. Given the endemicity of infections such as malaria, HIV, and tuberculosis (TB) in sub-Saharan Africa (SSA), it is important to evaluate coinfections with SARS-CoV-2 and their impact on maternal/infant outcomes. Robust research is critically needed to evaluate the effects of the added burden of COVID-19 in pregnancy, to help develop evidence-based policies toward improving maternal and infant outcomes. In this perspective, we briefly review current knowledge on the clinical features of COVID-19 in pregnancy; the risks of preterm birth and cesarean delivery secondary to comorbid severity; the effects of maternal SARS-CoV-2 infection on the fetus/neonate; and in utero mother-to-child SARS-CoV-2 transmission. We further highlight the need to conduct multicountry surveillance as well as retrospective and prospective cohort studies across SSA. This will enable assessments of SARS-CoV-2 burden among pregnant African women and improve the understanding of the spectrum of COVID-19 manifestations in this population, which may be living with or without HIV, TB, and/or other coinfections/comorbidities. In addition, multicountry studies will allow a better understanding of risk factors and outcomes to be compared across countries and subregions. Such an approach will encourage and strengthen much-needed intra-African, south-to-south multidisciplinary and interprofessional research collaborations. The African Forum for Research and Education in Health’s COVID-19 Research Working Group has embarked upon such a collaboration across Western, Central, Eastern and Southern Africa.

Data on SARS-CoV-2 and associated COVID-19 in pregnancy and their effects on maternal and fetal/infant health are limited. This is especially true for sub-Saharan Africa (SSA), where there is coexisting and highly prevalent HIV, tuberculosis (TB), malaria, and malnutrition, and some of the poorest maternal and child health outcomes globally.^1,2^ As of December 5, 2020, the WHO African region had recorded 1,529,436 COVID-19 cases and 34,125 deaths (case fatality rate: 2.2%).^3^ To the best of our knowledge, there have been no published prospective or large-scale studies evaluating the impact of SARS-CoV-2 infection on pregnancy in SSA.^4^ Comorbidities, coinfections, and socioeconomic and health system inequalities in SSA may interact with and worsen COVID-19 among pregnant African women and contribute to already poor health outcomes among these women and neonates (Figure 1). There is an urgent need to conduct epidemiological surveillance and cohort studies that evaluate the burden and effects of COVID-19 in the region, particularly among pregnant women.

**Figure 1.**
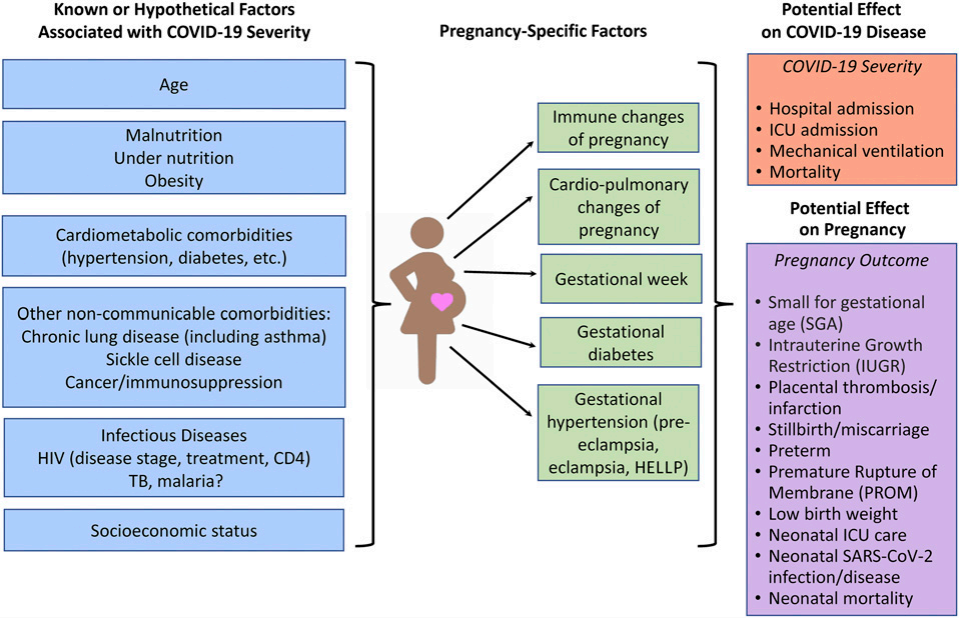
Known or hypothetical factors associated with COVID-19 disease, including pregnancy-specific factors, and effect on COVID-19 disease severity and potential effects on pregnancy outcome. HELLP = hemolysis, elevated liver enzymes, low platelets syndrome.

Other respiratory viral illnesses in pregnancy have been associated with high rates of adverse maternal and perinatal outcomes. For example, in the United States, 23% of pregnant women infected with influenza H1N1 required intensive care unit (ICU) admission, and 5% of all deaths due to H1N1 occurred among pregnant patients.^5,6^ Initial data on COVID-19 in pregnancy were derived from small case reports or case studies, first from China and then Italy and the United States, with most reporting on women infected during the third trimester; these initial reports suggested that pregnant women were not at higher risk for SARS-CoV-2 infection than nonpregnant women of similar age.^7–10^ Universal SARS-CoV-2 screening of women in labor in the United States and United Kingdom (UK) has shown that, as in the general population, a large proportion of SARS-CoV-2–infected pregnant women are asymptomatic.^9,11,12^ Among COVID-19 symptomatic women, fever and cough are the most observed symptoms in pregnancy.^10,13,14^ However, pregnant women who are asymptomatic at the time of delivery may be at risk for clinical progression in the postpartum period, developing fever and respiratory symptoms.^15,16^

Initial data discussed previously suggested that most pregnant women with COVID-19 had asymptomatic or mild disease. However, a recent evaluation of women of reproductive age with confirmed COVID-19 in the United States revealed that pregnant women were significantly more likely to be hospitalized and require ICU admission, mechanical ventilation, and extracorporeal membrane oxygenation (ECMO) than nonpregnant women with COVID-19, after adjusting for age, underlying conditions, and race/ethnicity.^7,14^ Nevertheless, the risk of mortality appears low and similar between pregnant and nonpregnant women of similar age (0.1–0.2%).^7,13,14^ As in the nonpregnant population, preexisting comorbidities, higher maternal age, and higher body mass index are risk factors for severe disease.^7,10,13,14^ There have been reports of severe complications of COVID-19 among pregnant women, including cardiomyopathy, coagulopathy, need for ECMO, and death.^9,17^

To date, there have been few maternal/infant COVID-19 studies from SSA. In a small observational cohort of pregnant women admitted with COVID-19 in Cameroon, common presenting symptoms were fever, cough, and dyspnea; ultimately four of the 18 women died in hospital (case fatality rate: 22%).^18^ Among the 13 women who delivered during the study, eight underwent cesarean section mostly because of maternal distress, and four newborns of the 13 deliveries died in hospital; however, neonatal outcomes could not be irrefutably attributed to COVID-19.^18^ A second small-scale study from Senegal reported on nine pregnant women presenting largely with cough and rhinorrhea who were admitted; median time to recovery was ∼14 days, and none of them died.^19^ Last, Nachega et al.^20^ described nearly 800 COVID-19 hospital admission cases from the Democratic Republic of the Congo, which included 12 pregnant women of a total of 262 women in the analysis. Three (25%) of the pregnant women had severe disease; however, none of the 12 pregnant women died, and there were also no significant differences in severity of disease between pregnant and nonpregnant women in this COVID-19 cohort. Of note, the last two studies did not report data on postpartum maternal or neonatal outcomes.

The risk of preterm birth and cesarean delivery secondary to maternal disease severity appears increased in pregnant women with COVID-19, compared with those without COVID-19, and neonates appear more likely to be admitted to the neonatal ICU (although in some cases solely for observation of SARS-CoV-2 exposure or secondary to prematurity).^10,13,21,22^ In general, beyond complications of prematurity, neonatal outcomes for infants born to mothers with COVID-19 appear good, and reports of COVID-19–attributable neonatal mortality are rare.^13,22^ These studies are primarily from China and high-resource countries such as the United States and United Kingdom. No study has yet addressed the effect of maternal COVID-19 in the first trimester on the developing fetus. There are a few case reports of miscarriage with SARS-CoV-2 infection in early pregnancy, but there are also reports of women with infection early in gestation who recover and have healthy, term infants.^23–25^

Data on the effects of maternal SARS-CoV-2 infection on the fetus/neonate are limited, partly because of the lack of collection of appropriate laboratory specimens. In utero mother-to-child transmission of SARS-CoV-2 appears rare.^26^ In a meta-analysis of 176 published cases of laboratory-confirmed SARS-CoV-2 infection in neonates, 12.2% of infections were thought to have occurred in utero (with only 5.7% having sufficient information to be classified as confirmed), 17.3% peripartum (with only 3.3% confirmed), and the majority of cases (70.5%) were classified as postnatal infection because of environmental exposure.^26^ Determination of in utero mother-to-child SARS-CoV-2 transmission requires appropriate samples obtained with proper timing, including amniotic fluid, placenta, neonatal blood, and nasopharyngeal and other samples from the infant at birth. Persistence of infection in the neonate should also be documented. However, collection of the needed tissues and fluids and/or data on timing of infant testing has not been provided in many studies to date and may not be routinely collected during clinical care, particularly in low- and middle-income countries because of limited laboratory testing capacity and other logistical challenges. In the Raschetti et al.^26^ meta-analysis, neonatal symptoms were observed in only 55% of SARS-CoV-2 PCR-positive neonates, and it may be difficult to distinguish symptoms because of prematurity from those due to SARS-CoV-2; there were only three neonatal deaths (2.5%), none of which were secondary to SARS-CoV-2 infection. Although SARS-CoV-2 has been on rare occasion identified by RT-PCR in breast milk, SARS-CoV-2 antibodies also have been documented, but the infectivity of the virus in breast milk has not been confirmed.^27–29^ Transmission of SARS-CoV-2 via breast milk has not been demonstrated to date.^28^

Although there are a number of registries that will target evaluation of the effects of SARS-CoV-2 infection in pregnant women and their neonates, they are predominantly in high-income settings (e.g., the UK Obstetric Surveillance System; the Pregnancy Coronavirus Outcomes Registry (PRIORITY) in the United States; the “MotherToBaby Pregnancy Studies” in the United States and Canada; the International Registry of Coronavirus Exposure in Pregnancy led by the United States and United Kingdom); none are focused on SSA.^23,30–32^ Given the paucity of data on SARS-CoV-2 and pregnancy from SSA, there is an urgent need to conduct surveillance and prospective studies across the region. Furthermore, retrospective analyses of routinely collected data are important, to assess the burden of SARS-CoV-2 infection among pregnant women (likely reflecting disease burden in the community) and to understand whether and how COVID-19 manifests differently among African women with HIV, TB, malaria, and other coinfections/comorbidities endemic to SSA. Comparisons of risk factors and outcomes can also be made across SSA countries and subregions while strengthening much-needed intra-African south-to-south multi-disciplinary and interprofessional research collaborations. The African Forum for Research and Education in Health’s^33^ COVID-19 Research Working Group has embarked upon such a collaboration across Western, Central, Eastern, and Southern Africa.

Finally, despite the fact that pregnant women appear to be at higher risk of morbidity and mortality from COVID-19 than age-matched, nonpregnant women, pregnant women have been excluded from SARS-CoV-2 vaccine trials to date.^34^ Two SARS-CoV-2 mRNA-based vaccines have been reported to have high efficacy in reducing infections, with both already having received emergency use authorization by the UK Medicines and Healthcare Regulatory Agency and the U.S. Food and Drug Administration.^35,36^ Given the preponderance of women of reproductive age who work in health care (particularly in SSA), an area of profession that will be prioritized for initial phases of vaccine implementation, it is likely that some of these women will be pregnant, and others may be pregnant but not know it at the time of vaccination. Systems will need to be developed to track maternal/infant outcomes of COVID-19 vaccination in such pregnant women, which will be particularly challenging in SSA settings with weak health (especially in pregnancy) monitoring systems. As COVID-19 vaccines are rolled out globally, it is important to develop collaborative partnerships to enable surveillance of maternal/infant outcomes in SSA.

In the context of a global pandemic, vulnerable populations such as pregnant women and their infants are often neglected. This was observed in the initial years of the HIV epidemic and again with the Ebola virus, when clinical trials for drug and vaccine interventions for these high-fatality diseases did not include pregnant women, despite the high mortality in women and their infants.^37^ It is critical during the SARS-CoV-2 pandemic that we learn from prior experiences and include pregnant women and their infants in studies to 1) better understand the extent of infection and disease in this population and the effects on maternal/child health and 2) ensure consideration of pregnant women, as new treatments and vaccines to combat SARS-CoV-2 are being developed.
